# Developmental coupling of larval and adult stages in a complex life cycle: insights from limb regeneration in the flour beetle, *Tribolium castaneum*

**DOI:** 10.1186/2041-9139-4-20

**Published:** 2013-07-04

**Authors:** Alison K Lee, Christie C Sze, Elaine R Kim, Yuichiro Suzuki

**Affiliations:** 1 Department of Biological Sciences, Wellesley College, 106 Central St., Wellesley, MA 02481, USA

**Keywords:** Limb regeneration, Distal-less, Dachshund, Spineless, Abrupt, *Tribolium castaneum*, Patterning, Blastema

## Abstract

**Background:**

A complex life cycle, such as complete metamorphosis, is a key innovation that can promote diversification of species. The evolution of a morphologically distinct larval stage is thought to have enabled insects to occupy broader ecological niches and become the most diverse metazoan taxon, yet the extent to which larval and adult morphologies can evolve independently remains unknown. Perturbation of larval limb regeneration allows us to generate larval legs and antennae with altered limb morphologies, which may be used to explore the developmental continuity that might exist between larval and adult appendages. In this study, we determined the roles of several appendage patterning transcription factors, *abrupt* (*ab*), *dachshund* (*dac*), *Distal-less* (*Dll*), and *spineless* (*ss*), in the red flour beetle, *Tribolium castaneum,* during larval appendage regeneration. The functions of these genes in regenerating and non-regenerating limbs were compared using RNA interference.

**Results:**

During limb regeneration, *dac* and *ss* were necessary to re-pattern the same larval structures as those patterned during embryogenesis. Removal of these two genes led to larval appendage patterning defects that were carried over to the adult legs. Surprisingly, even though maternal knockdown of *ab* had minimal effects on limb allocation and patterning in the embryo, it was necessary for blastema growth, an earlier phase of regeneration. Finally, knockdown of *Dll* prevented the blastema-like bumps from re-differentiating into appendages.

**Conclusions:**

Our results suggest that, similar to vertebrates, the re-patterning phase of *Tribolium* larval limb regeneration relies on the same genes that are used during embryonic limb patterning. Thus, the re-patterning phase of regeneration is likely to be regulated by taxon-specific patterning mechanisms. Furthermore, Ab and Dll appear to play important roles during blastema proliferation and re-differentiation, respectively. Finally, our results show that continuity exists between larval and adult limb patterning, and that larval and adult leg morphologies may be developmentally coupled. Thus, the evolution of imaginal discs may have been a key step towards completely removing any developmental constraints that existed between larval and adult phenotypes.

## Background

Complex life cycles, which contain two or more morphologically discrete postembryonic phases, are found ubiquitously among many metazoan taxa [1]. Such complex life cycles can serve as adaptive strategies that allow different developmental stages to evolve independently to occupy larger ecological niches [1]. For example, holometabolous insects, or insects that undergo complete metamorphosis, produce distinct larval and adult morphologies that are capable of freely adapting to different ecological habitats. The evolution of two morphologically distinct stages is thought to be a key innovation that greatly aided the diversification of these insects [1,2]. However, whether or not the larval morphology is completely free to evolve without influencing the adult phenotype remains unclear.

The molecular mechanisms underlying the transition from larval to adult stages are well-understood, yet the cellular origins of the adult body remain an enigma. In higher dipterans and a number of other insect species, clusters of cells form a distinct tissue called an imaginal disc, which develops inside the larva. In fruit flies, much of the adult body forms from these imaginal discs, and the larval cells make limited contributions to the adult body [3]. However, in most other insects, adult structures do not arise from free-floating imaginal discs, but rather develop from cells associated with larval structures. In lepidopterans, for example, imaginal cells embedded within the larval tissues replace much of the larval-specific cells to generate the adult structures [4,5]. In other insects, such as beetles, polymorphic larval cells are thought to make direct contribution to the developing adult body [5,6], although more studies are needed before definitive conclusions can be drawn.

Given that most insects do not make their entire adult body from set-aside imaginal discs, the larval morphology likely imposes some constraint on the development of the adult morphology. To determine the extent to which various stages of a complex life cycle can independently evolve, an understanding of the developmental relationships among the different stages is essential. In this study, we examined the consequence of genetic perturbations during larval appendage regeneration, to examine the degree of developmental continuity that might exist between larval and adult morphologies.

We chose to work with a model holometabolous insect, *Tribolium castaneum,* whose legs and antennae undergo two major morphogenetic transitions during the life cycle: 1) embryonic to larval appendages during embryogenesis, and 2) larval to adult appendages during metamorphosis [7]. Knockdown studies in *Tribolium* have shown that the embryonic patterning of larval legs and the metamorphic patterning of adult legs rely on overlapping but distinct sets of genes [8-11]. Because embryonic gene knockdown often leads to lethality of the animal due to pleiotropic side-effects caused by gene silencing, it is difficult to know how alterations of larval limb morphologies during embryogenesis affect the adult morphology. Thus, in order to assess the continuity between larval leg and adult leg patterning, the larval appendage morphology was altered by molecular perturbations during regeneration, to assess the potential impact on the regenerated adult morphology. *Tribolium* is well suited for genetically perturbing regeneration, given its short life cycle, fully sequenced genome [12], and amenability to functional analyses using RNA interference (RNAi) [13]. Importantly, it develops external larval appendages, such as legs and antennae, which are capable of complete regeneration in a relatively short amount of time [14]. The larvae are capable of regenerating their appendages, and within two molts, much of the larval appendage morphology is restored. Once they become a pupa or an adult, *Tribolium* do not regenerate their appendages. By disrupting larval leg re-patterning, we can begin to address how larval leg patterning and adult leg patterning might be linked.

In this study, we knocked down the expression of four transcription factors that have been previously identified as key regulators of normal appendage patterning: *dachshund* (*dac*), *Distal-less* (*Dll*), *abrupt* (*ab*) and *spineless* (*ss*). *dac* codes for a transcription factor required for development of the mid-limb segment along the proximal-distal (PD) axis in *Drosophila* and *Tribolium*[15,16]*.* In *Manduca*, its embryonic expression likewise suggests a conserved role in mid-limb segment patterning [15-17]. In *Tribolium*, Dac has been determined to play a critical role in both adult mid-leg segment patterning [9,18] and adult antennal segmentation [8]. *dac* expression in *Tribolium* has been analyzed in the embryonic stage [15], but whether it has a major function in patterning larval legs or antennae during this stage is not known.

*Dll* is a limb-patterning gene encoding a homeodomain transcription factor required for distal limb development in insects [8,9,18-20]. In *Tribolium, Dll* is required for proper leg patterning along the PD axis during embryonic development [20] and metamorphosis [9,18]. *Dll* has also been established to be important for antenna segmentation in the developing *Tribolium* pupa [8] and embryo [20].

In addition to Dac and Dll, several additional transcription factors have also been identified to be important for patterning limbs during metamorphosis [8,9]. We chose to investigate the role of two of these transcription factors during regeneration: the Bric-a-brac Tramtrack Broad complex (BTB) protein, Ab, and the basic-helix-loop-helix-PAS transcription factor, Ss. Ab is a BTB-zinc finger transcription factor necessary for antennal and tarsal segmentation in *Tribolium.* Knockdown of *ab* in *Tribolium* larvae results in a reduction of the number of adult antennal segments [8] and fusion of tarsomeres of the adult legs [9]. During postembryonic development of *Drosophila*, Ab is involved in establishing and maintaining muscle attachments and morphogenesis of adult appendages: viable *ab* mutants have deformed antennae and bristles, gnarled legs and wing venation defects [21-23]. Its role during embryonic limb development is not known although it has been identified as a regulator of neuromuscular junction development in *Drosophila*, where it is expressed in muscle cells but not neurons [21,24].

Ss is also required for leg segmentation in *Tribolium* and *Drosophila* during metamorphosis [9,25]. Knockdown of *ss* in *Tribolium* results in the development of adult legs with fused tarsomeres. In addition, homeotic transformations of the distal portions of the adult antennae into a tibia-like segment with a terminal claw result from *ss* knockdown during the larval stage [8,10]. Similarly, in *Drosophila,* Ss is required for specifying antennal identity, and *ss* mutants exhibit antenna-to-leg transformations [25-28]. During *Tribolium* embryonic antennal development, Ss plays a critical role in establishing larval antennal identity [10,29]. Silencing of *ss* during embryogenesis results in the formation of newly hatched larvae with legs instead of antennae [10,29]. However, its removal does not appear to influence embryonic leg development; thus, embryos develop larval legs normally even when *ss* is silenced, while *ss* knockdown during metamorphosis results in adults with fused tarsomeres.

We hypothesized that the removal of patterning genes required for larval limb development would also be required for larval limb regeneration. We also hypothesized that disrupting the larval limb patterning during regeneration would influence the adult limb patterning and illustrate the continuity that exists between larval and adult limb patterning. Thus, we determined the phenotypes of embryonic appendages resulting from silencing Dac and Ab, and the effects of *dac*, *Dll*, *ab* and *ss* knockdowns during larval limb regeneration in *Tribolium*. We found that both Ab and Dll are necessary for limb regeneration prior to patterning, suggesting regeneration-specific functions of these genes. We also found that perturbed patterning of larval limbs leads to corresponding alterations in the adult limb morphology, indicating the continuity of patterning across larval and adult structures.

## Methods

### Beetle husbandry

*Tribolium casteneum* strain GA-1 was obtained from Dr Richard Beeman (USDA ARS Biological Research Unit, Grain Marketing & Production Research Center, Manhattan, KS, USA). Beetles were raised on organic wheat flour containing 5% nutritional yeast, and stocks were maintained in plastic containers in a 29°C incubator with approximately 50% relative humidity. No ethical approval was necessary to work with *Tribolium* as they are common household insect pests.

### mRNA isolation and cDNA synthesis

Larvae were dissected in 1X-PBS (0.02 M phosphate, 0.15 M NaCl, 0.0038 M NaH_2_PO_4_, 0.0162 M Na_2_HPO_4_; pH 7.4) to remove the gut and the fat body. The remaining tissue was homogenized in Trizol (Invitrogen, Carlsbad, CA, USA), and RNA was subsequently extracted using chloroform, treated with DNase (Promega, Fitchburg, WI, USA) and precipitated in isopropyl alcohol. cDNA was synthesized from 1 μg of total RNA using the cDNA synthesis kit (Thermo Fisher Scientific, Pittsburgh PA, USA) following the manufacturer’s instructions.

### Cloning and double-stranded RNA (dsRNA) synthesis

Sequences of *Dll* [AF317551], *dac* [XM_964678], *ab* [XM_969854] and *ss* [EU912437] were obtained from confirmed sequences deposited in GenBank [8,10,18], and primers were designed outside of highly conserved regions to avoid off-target knockdown of gene expression. The list of primers used for cDNA amplification is given in Table 1. The amplified cDNA product was isolated and subsequently cloned into a TOPO TA vector (Invitrogen, Carlsbad, CA, USA). Following plasmid identity confirmation by sequencing, plasmid DNA was linearized via restriction digestion.

**Table 1:** **Primers used for*ab, dac, Dll*and*ss*dsRNA synthesis and knockdown verification**

**Gene**	**Application**	**Forward primer (5′->3′)**	**Reverse primer (5′->3′)**
*ab*	RNAi	ACTCACAAAGGAGAAGGGAAA	CGTTGGTATTGAAAGGATGG
	Knockdown verification	GCCTGTGATGGATGTTCGT	GTCTTGGGTCTGTCGCTCT
*ss*	RNAi	ATTACTCAAAACTGGCGCTTC	TGTTGTGTTAGTGGGAGGAGTT
	Knockdown verification	GGAGATACCGCAGAAGGAAG	TTGTAGTCAGGGGCGATGT
*dac*	RNAi	CAGCATCGCATCTTCAAC	CTCCTCCCTCAGCCTTTCT
	Knockdown verification	ACTGCACTACGGCCAGTTC	TCGTCCATGTCTTGATCCTT
*Dll*	RNAi	GGATAACAAACCCTTCACGAC	GCCTCTCCAACGATAAACAC
	Knockdown verification	GGTGTGTTCGTAGTGCTTCC	CGCCTTCATCATCTTCTTGT

RNAi, RNA interference; *ab*, *abrupt*; *ss*, *spineless*; *dac*, *dachshund*; *Dll*, *Distal-less.*

Each of the strands of the dsRNA was synthesized with the T3 and T7 MEGAscript Kits (Ambion) following the manufacturer’s instructions. Single-stranded RNA (ssRNA) was combined and annealed to form dsRNA [30]. The annealed product was analyzed by gel electrophoresis to confirm annealing, and stored at −80°C until use. The final concentrations of the dsRNA were 2 μg/μL for *ab*, *dac*, *Dll* and *amp*^*r*^ (plasmid gift from Dr Takashi Koyama, Gulbenkian Institute, Lisbon), and 2 μg/μL or 4 μg/μL for *ss*.

### DsRNA injection

Approximately 0.5 μg (0.25 μL) of dsRNA was injected into the dorsal side of the day-0 fifth or sixth instar larvae (the final instar in our colony is typically the seventh or eighth instar), between the first and second abdominal segments, with a pulled 10-μL glass capillary needle connected to a syringe. Control larvae were injected with the same amount of bacterial *amp*^*r*^ dsRNA. To visualize the effects of dsRNA on developing embryos, parental RNAi was conducted on female *Tribolium* adults [20]. Approximately 0.5 μL of dsRNA was injected into the abdominal body cavity of the adult female insects. The eggs from the dsRNA-injected female adults were separated out from the flour every 4 to 5 days, starting one week after dsRNA injection. The eggs were maintained at 29°C for another 4 to 5 days to allow the eggs to develop. The developing unhatched embryos or early hatched larvae (first instar) were then processed with lactic acid to clear the egg shell and the hard cuticle for fluorescent microscopy as previously described [31,32]: unhatched embryos were incubated in 75% lactic acid, while hatched larvae were incubated overnight at 60°C in 10% ethanol: 90% lactic acid solution. These were then stored in 70% ethanol: 15% glycerol solution, or mounted in 80% glycerol. Embryos were imaged and scored using fluorescence microscopy.

### Leg and antennal ablations

Two days after dsRNA injections, the larval mid- and hindlegs were ablated at the coxa on one side [14]. The larvae were placed on double-sided tape after anesthesia, and the legs were ablated using a pair of microscissors. The forelegs and the contralateral mid- and hindlegs served as internal controls for analyzing the effect of each dsRNA on regeneration of larval and adult legs. The larval antenna from the left side of the head was ablated using a razor blade two days after dsRNA injection. Anesthetized larvae were placed ventral side uppermost on double-sided tape, and the antennae were sliced off using the razor blade. The contralateral antenna served as the internal control. Larvae were stored in a 70% ethanol: 15% glycerol solution until imaged. The legs and heads were mounted in 80% glycerol and imaged using light and fluorescence microscopy, respectively.

### Knockdown verification

To determine whether gene expression was knocked down in response to dsRNA injection, gene expression was analyzed using semiquantitative PCR. Day-0 sixth-instar larvae were injected with *dac, Dll, ab*, *ss* or control *amp*^*r*^ dsRNA. For each treatment, two days after molting into the seventh instar, three larvae were pooled in Trizol and homogenized to isolate their RNA. After DNAse treatment, 1 μg of RNA was converted to cDNA and amplified using semiquantitative PCR. The PCR was repeated until the optimum cycle numbers were obtained, such that the bands were unsaturated. The following cycle numbers were used: *dac* = 42; *Dll* = 39; *ab* = 38; *ss* = 40; *rp49* = 34.

## Results

### Embryonic functions of *dac* and *ab*

We first report the effects of maternal *dac* and *ab* dsRNA injection on embryonic appendage development, since knockdown embryonic phenotypes for these genes have not yet been reported. The results of maternal RNAi are summarized in Table 2. *Dll* and *ss* knockdown produced phenotypes similar to those reported previously (see Additional file 1 and Tables 2 and 3) [10,20,29,33].

**Table 2:** Summary of the knockdown effects observed in this study

**Appendage**	**Gene**	**No ablation**			**Ablation**
		**Maternal RNAi**	**Larval RNAi**		**Larval RNAi**
		**Larva**	**Larva**	**Pupa/adult**	**Larva**
Leg	*Dll*	Loss of distal leg segments	Loss of tissue identity and regression	Loss of tissue identity and regression	Bump formation and no outgrowths
	*dac*	Partial fusion of femur and tibiotarsus	No effect	Loss of distal femur and tibia and fusion of proximal tarsomeres	Partial fusion of femur and tibiotarsus
	*ab*	Slightly compressed but segment number is unaffected	No effect	Partial loss of tarsomeres	Small bump formation and no outgrowths
	*ss*	No effect	No effect	Fusion of tarsomeres	No effect
Antenna	*Dll*	Loss of distal antennal segments	Loss of tissue identity	Partial transformation into a leg	Bump formation and no outgrowths
	*dac*	No effect	No effect	Partial loss of antennal segments	No effect
	*ab*	Slightly compressed, but segment number is unaffected	No effect	Partial loss of antennal segments	No outgrowths in some larvae; others no effect
	*ss*	Partial transformation into a leg	No effect	Partial transformation into a leg	Partial transformation into a leg

The leg and antennal phenotypes observed after maternal RNA interference (RNAi) or larval RNAi of *ab, dac, Dll* and *ss* expression with and without ablation are summarized. *Dll*, *Distal-less*; *dac*, *dachshund*; *ab*, *abrupt*; *ss*, *spineless*.

**Table 3:** **Summary of the effects of *ab*, *dac*, *Dll *and *ss *dsRNA injection on embryonic appendage development**

**Treatment (dsRNA)**	**Total number of embryos**	**Antenna**			**Leg**		
		**Unaffected**	F/Sc/T*	**Short**	**Unaffected**	F/Sc/T*	**Short**
*amp*^*r*^	**49**	49 (100%)	0 (0%)	0 (0%)	49 (100%)	0 (0%)	0 (0%)
*dac*	**47**	47 (100%)	0 (0%)	0 (0%)	23 (49%)	24 (F)(51%)	0 (0%)
*Dll*	**53**	4 (8%)	0 (0%)	49 (92%)	4 (8%)	0 (0%)	49 (92%)
*ss*	**58**	0 (0%)	58 (T)(100%)	0 (0%)	58 (100%)	0 (0%)	0 (0%)
*ab*	**65**	7 (11%)	57 (Sc)(88%)	1 (1%)	7 (11%)	58 (Sc)(89%)	0 (0%)

*F/Sc/T denotes embryos that are fused (F), scrunched (Sc) or transformed (T). *amp*^*r*^*, ampicillin-resistance; dac*, *dachshund*; *Dll*, *Distal-less*; *ss*, *spineless*; *ab*, *abrupt*.

Eggs collected from female beetles injected with *ab, dac, Dll* and *ss* dsRNA (2 μg/μL) of were collected and processed to determine the phenotypes of the developing embryonic appendages, the antennae and legs. The *amp*^*r*^-treated embryos were used as the control. The antenna and legs were scored according to the following categories: unaffected, F/Sc/T (fused, scrunched, or transformed compared to the control), or short (lengths of segments are different from the control). *amp*^*r*^, *ampicillin*-*resistance*; *dac*, *dachshund*; *Dll*, *Distal-less*; *ss*, *spineless*; *ab*, *abrupt.*

**Figure 1:**
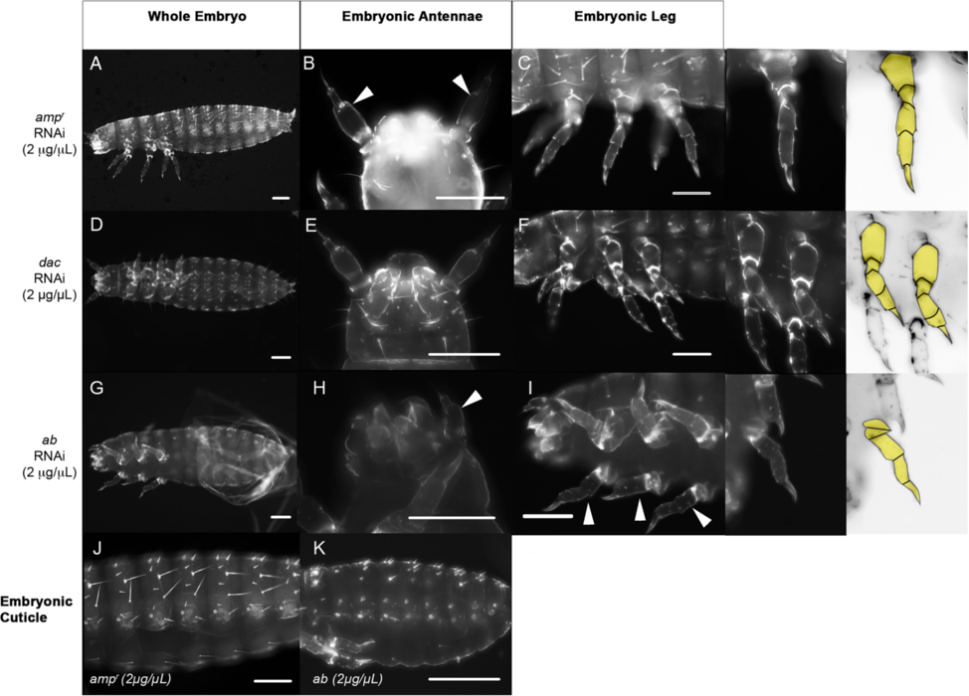
**Effects of patterning gene knockdown on embryonic appendage development.** Eggs and newly hatched larvae from female beetles injected with of 2 μg/μL *ampicillin-resistance* (*amp*^*r*^) **(A**-**C)***, dachshund* (*dac*) **(D**-**F)** and *abrupt* (*ab*) **(G**-**I)** double-stranded (ds)RNA were collected and processed to compare the phenotypes of resulting antennae and legs. Newly hatched *amp*^*r*^ dsRNA-treated larvae were used as a control. The antennae **(B**,**E**,**H)** and legs **(C**,**F**,**I)** were visualized under fluorescence microscopy and are marked with arrowheads. Right panel shows a color-level inversion image with the leg segments highlighted in yellow. **(J)** Lateral view of the abdomen of a newly hatched larva obtained from female adults injected with *amp*^*r*^ dsRNA. **(K)** Lateral view of the abdomen of the late embryo obtained from female adults injected with *ab* dsRNA. Scale bars represent 0.1 mm.

All of the *dac* knockdown embryos (n = 47) (Table 3) developed normal antennal morphology (Figure 1E), similar to antennae of the control *amp*^*r*^ dsRNA-treated embryos (Figure 1B). In contrast, 49% of the *dac* dsRNA-injected embryos (Table 3) had partially fused femur and tibiotarsus (Figure 1D and 1F), which were not seen in the *amp*^*r*^ dsRNA-injected control embryos (Figure 1A and 1C). The coxa and trochanter, however, were unaffected in size or segmentation*.* Thus, *dac* knockdown caused a reduction in size of the femur-tibiotarsal leg segments, whereas the antennae remained unaffected (Figure 1E and 1F).

The majority of *ab* knockdown embryos produced through maternal RNAi exhibited an abnormal scrunched phenotype (Table 3; Figure 1G-I), where the body and appendages all appeared broader and compressed. The antenna (n = 57) and legs (n = 58) of the *ab*-knockdown embryos both exhibited this characteristic scrunched phenotype, where the cuticle failed to be fully extended. However, these appendages had the typical number of segments in the right proportions. This suggests that Ab does not affect the structural formation or patterning of these appendages in the embryo.

Besides the scrunched phenotype, *ab* knockdown dramatically decreased the number of bristles that were present on the surface of the embryonic cuticle. Compared to the *amp*^*r*^ dsRNA-injected embryos (Figure 1J), there were fewer bristles present on the dorsal tergites of *ab* knockdown embryos (Figure 1K). This suggests that Ab is important for embryonic bristle formation and cuticular expansion, but not for appendage segmentation or patterning.

### Knockdown of *dac* expression prevents regeneration of the medial segments of legs

**Figure 2:**
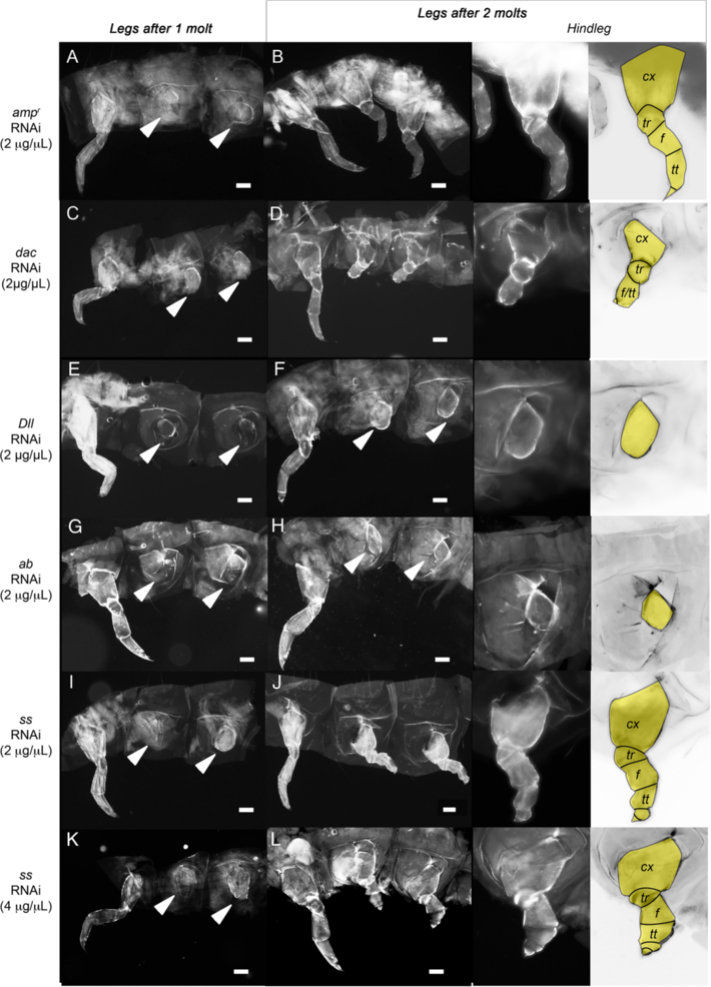
**The effect of patterning gene knockdown on *Tribolium *larval leg regeneration one or two molts after leg ablation*. *(A**,**C**,**E**,**G**,**I**,**K)** Regenerating larval legs after one molt in larvae injected with *ampicillin-resistance* (*amp*^*r*^) double-stranded (ds)RNA **(A)**, *dachshund* (*dac)* dsRNA **(C)**, *Distal-less* (*Dll)* dsRNA **(E)**, *abrupt* (*ab)* dsRNA **(G)**, *spineless* (*ss)* dsRNA (2 μg/μl) **(I)** and *ss* dsRNA (4 μg/μl) **(K)**. **(B**,**D**,**F**,**H**,**J**,**L)** Regenerating larval legs after two molts in larvae injected with *amp*^*r*^ dsRNA **(B)**, *dac* dsRNA **(D)**, *Dll* dsRNA **(F)**, *ab* dsRNA **(H)**, *ss* dsRNA (2 μg/μl) **(J)** and *ss* dsRNA (4 μg/μl) **(L)**. Arrowheads indicate the bases of the ablated legs. Middle panel shows the close-up image of the hindleg. Right panel shows a color-level inversion image with the regenerating leg segments highlighted in yellow. *Cx,* coxa; *tr*, trochanter; *f*, femur; *tt*, tibiotarsus. Scale bars correspond to 0.1 mm.

**Figure 3:**
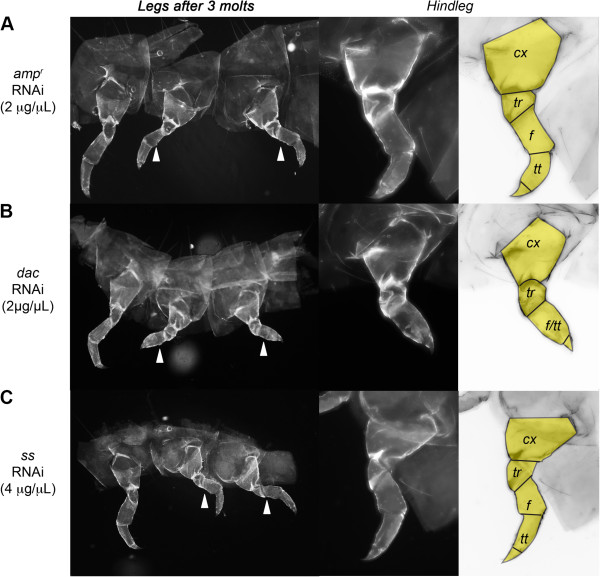
**Regenerated legs of larvae after three molts post gene knockdown and leg ablation.(A**-**C)** Regenerating larval legs injected with *ampicillin-resistance* (*amp*^*r*^) double-stranded (ds)RNA **(A)**, *dachshund* (*dac)* dsRNA **(B)** and *spineless* (*ss)* dsRNA (4 μg/μl) **(C)**. The mid- and hindlegs (marked with arrowheads) were removed. A close-up of the hindleg is shown in the center, and right panels show a color-level inversion image with the regenerated leg segments highlighted in yellow. Fourth-instar larvae were injected on day 0, and legs were removed on day 2.

We next examined the effects of larval RNAi during regeneration. All of the phenotypic effects observed in this study are summarized in Table 2. To examine the role of Dac during *Tribolium* leg regeneration, *dac* dsRNA was injected into day-0 fifth- or sixth-instar larvae. Two days later, the mid- and hindlegs on one side of these larvae were ablated, and the regeneration of the ablated appendage after every molt was recorded (Table 4). After one molt, the site of ablation was completely healed in all larvae (n = 18) (Table 4; Figure 2A and C). After two molts, all larvae (n = 16) began to regenerate truncated legs (Table 4). The regenerating mid- and hindlegs of the *dac* dsRNA-injected animals had regenerated the coxa, trochanter and distal claw, but the femur and tibiotarsal segment appeared to be shortened (Figure 2D) compared to the *amp*^*r*^ dsRNA-injected regenerating limbs (Figure 2B). When smaller larvae were injected, the larvae molted three times, allowing us to see the effect of *dac* knockdown on the regenerating larval leg. When *amp*^*r*^ dsRNA-injected larvae molted three times, the morphology of all segments was restored (Figure 3A). In contrast, when *dac* expression was knocked down, the femur and tibiotarsus of the regenerated legs were partially fused (Figure 3B). This leg phenotype was similar to the embryonic legs of *dac*-knockdown larvae (compare Figures 1F and 3B).

**Table 4:** **Summary of effects of patterning gene knockdowns on larval leg regeneration in *Tribolium***

			**Larvae after 2 molts**			**Pupae after 1 molt**		**Pupae after 2 molts**	
**dsRNA injected**	**Concentration**	**Total number**	**Number with regenerated legs**	**Number with no regenerated legs**	**Died before pupation after 2 larval molts**	**Number with leg regeneration**	**Number with no leg regeneration**	**Number with leg regeneration**	**Number with no leg regeneration**
*amp*^*r*^	**2 μg/μL**	23	10	0	3	13	0	7	0
*dac*	**2μg/μL**	18	16*	0	4	2	0	12*	0
*Dll*	**2μg/μL**	18	0	18	16	0	0	0	2
*ab*	**2 μg/μL**	28	0	19	13	0	9	0	6
*ss*	**4 μg/μL**	18	11	0	3	7	0	8	0
	**2 μg/μL**	10	7	0	1	3	0	6	0

The fifth- or sixth-instar larvae were injected with *amp*^*r*^, *dac*, *Dll*, *ab* and *ss* dsRNA, and their legs were ablated. *amp*^*r*^ dsRNA injections served as controls. *Regeneration in the *dac* dsRNA-injected insects was different from that in the control *amp*^*r*^ dsRNA-injected insects. *amp*^*r*^, *ampicillin*-*resistance*; *dac*, *dachshund*; *Dll*, *Distal-less*; *ab*, *abrupt*; *ss*, *spineless.*

**Figure 4:**
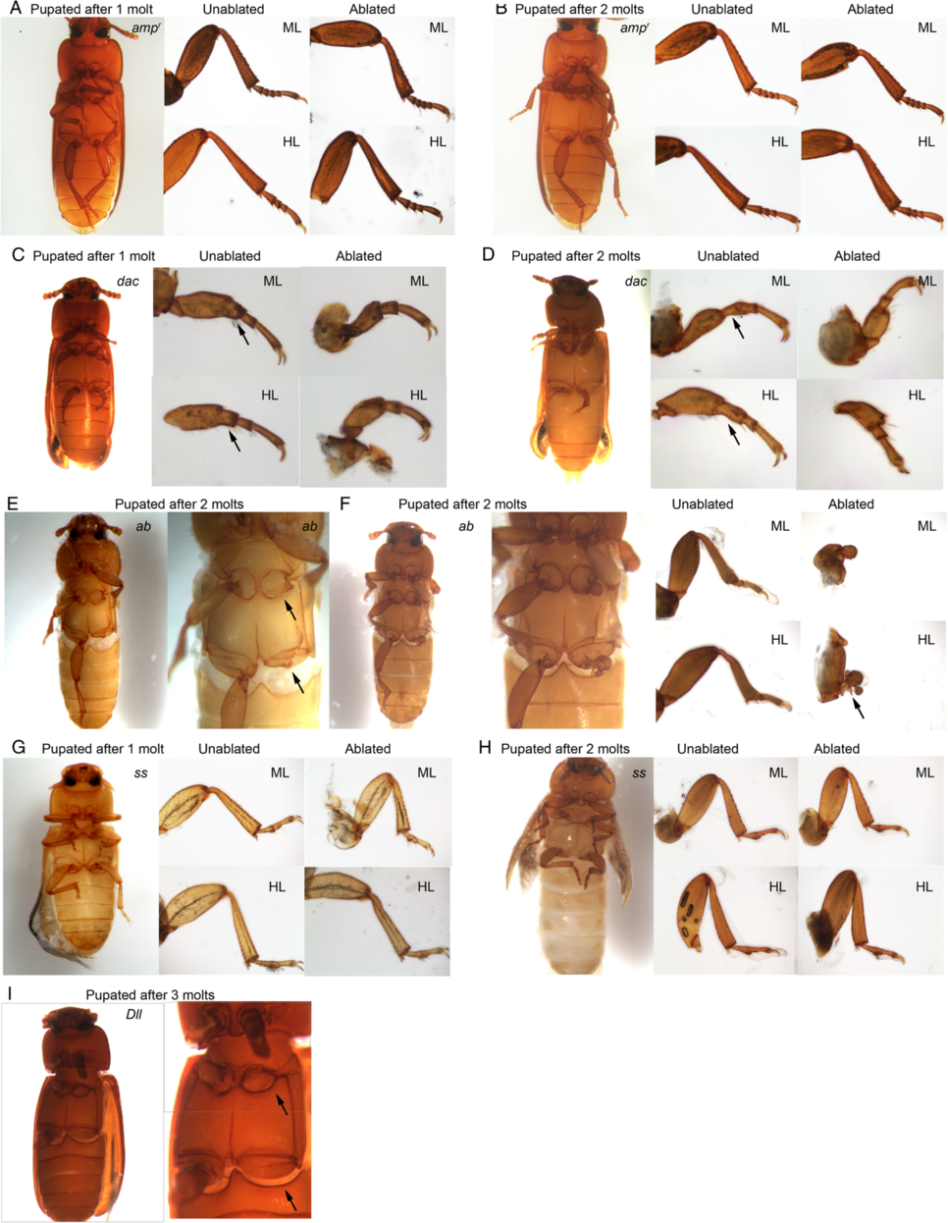
**The effects of appendage patterning gene knockdowns on adult leg regeneration in *Tribolium*. (A, B)** Adult leg phenotype of larvae injected with 2 μg/μL *ampicillin-resistance* (*amp*^*r*^) double-stranded (ds)RNA. Adults formed from pupae that experienced one molt **(A)** or two molts **(B)** after dsRNA injection. Close-up of the unablated (middle) and ablated (right) mid- (top) and hindlegs (bottom) are shown. **(C**,**D)** Adults that formed after larvae were injected with 2 μg/μL *dachshund* (*dac)* dsRNA and underwent one molt **(C)** and two molts **(D)** before pupation. Close-up of the unablated (middle) and ablated (right) mid- (top) and hindlegs (bottom) are shown. Arrowheads indicate the segment on the unablated leg that is missing in the ablated leg. **(E**,**F)** Adults that formed after larvae were injected with 2 μg/μL *abrupt* (*ab)* dsRNA and underwent two molts before pupation. Close-up of the unablated (middle) and ablated (right) mid- (top) and hindlegs (bottom) are shown. **(E)** Arrows indicate the site where the legs should have regenerated. **(F)** Arrow indicates the claw-like structure that developed at the base of the leg. **(G**,**H)** Adults that formed after larvae were injected with 4 μg/μL *ss* dsRNA and underwent one molt **(G)** and two molts **(H)** before pupation. Close-up of the unablated (middle) and ablated (right) mid- (top) and hindlegs (bottom) are shown. **(I)** Adults that formed after larvae were injected with 2 μg/μL *Distal-less* (*Dll)* dsRNA and underwent three molts before pupation. Arrows indicate the sites where the legs should have regenerated.

As reported previously, adults that developed after *dac* dsRNA-injection in the larval phase lacked the distal femur and tibia and had fused proximal tarsomeres [9,18]. When legs were ablated in these *dac* dsRNA-injected animals, the regenerated legs in adults that underwent two molts prior to metamorphosis had smaller femurs and fused tarsomeres, unlike those of the uncut contralateral legs (n = 12) (Table 4; Figure 4C and D). The femur and the segment corresponding to fused tarsomeres were reduced in length, and in some cases, were missing. These data indicate that Dac plays a role in re-patterning the medial segments during leg regeneration and that the alteration in larval leg patterning is carried over to the adult leg patterning.

**Figure 5:**
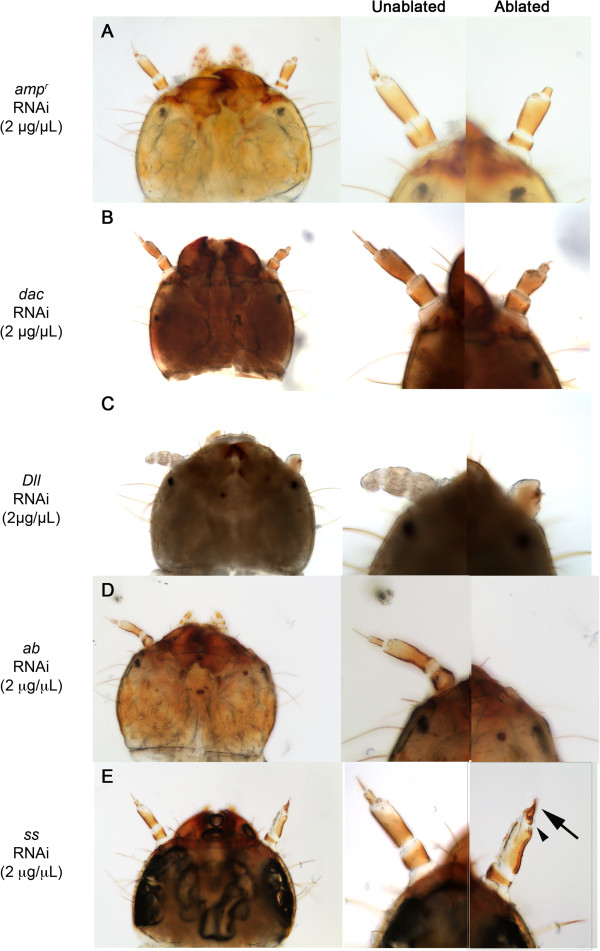
**Effects of patterning gene knockdown on larval antennal regeneration.** Larvae were treated with 2 μg/μL *ampicillin-resistance* (*amp*^*r*^) **(A)***, dachshund (dac)***(B)**, *Distal-less* (*Dll)***(C)**, *abrupt* (*ab)***(D)** and *spineless* (*ss)***(E)** double-stranded (dsRNA). Left panels, the head of the treated larvae; middle panels, unablated antennae; right panels, ablated antennae. **(E)** Arrow indicates the claw-like structure in the regenerated antenna of *ss* dsRNA-injected larva. The arrowhead indicates the ectopic bristle on the transformed antenna.

**Figure 6:**
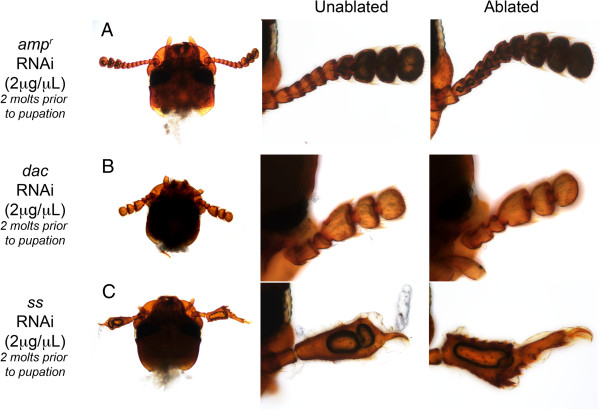
**Effects of patterning gene knockdown on adult antennal regeneration.** Adults that formed after larvae were treated with 2 μg/μL *ampicillin-resistance* (*amp*^*r*^) **(A)***, dachshund (dac)***(B)** and *spineless* (*ss)***(C)** double-stranded (ds)RNA. All injected animals underwent two molts prior to the prepupal stage. Comparable pictures were taken under the same magnification.

The silencing of *dac* expression during the larval stage did not appear to affect the regeneration of antennae (Table 5; Figure 5B). After two molts, all of the *dac* dsRNA-injected larvae (n = 14) regenerated their cut antennae. They either partially developed a new segment distal to the site of ablation, or fully regenerated their larval antennae, as observed in the *amp*^*r*^ dsRNA-injected animals (Figure 5B). All regenerated antennae were indistinguishable from the unablated contralateral antennae (Figure 6B) in all adults that pupated after two molts (n = 12), and both ablated and unablated antennae had reduced numbers of segments as reported previously [8,18]. This indicates that Dac does not play a major role during antennal regeneration, whereas it is necessary for proper antennal development during metamorphosis.

**Table 5:** **Summary of the effects following knockdown of patterning genes on larval antennal regeneration in *Tribolium***

			**Larvae after 2 molts**			**Pupae after 1 molt**		**Pupae after 2 molts**	
**dsRNA injected**	**Concentration**	**Total number**	**Number with regenerated antennae**	**Number with no regenerated antennae**	**Died before pupation after 2 larval molts**	**Number with antennal regeneration**	**Number with no antennal regeneration**	**Number with antennal regeneration**	**Number with no antennal regeneration**
*amp*^*r*^	**2 μg/μL**	14	10	0	0	4	0	10	0
*dac*	**2 μg/μL**	27	14	0	2	10	3	12	0
*Dll*	**2 μg/μL**	10	0	7	7	0	3	0	0
*ab*	**2 μg/μL**	14	4	7	11	2	1	0	0
	**0.5 μg/μL**	9	2	7	8	0	0	1	0
*ss*	**2 μg/μL**	22	21*	0	9	0	1	12*	0

The left antennae of fifth- and sixth-instar larvae treated with *abrupt* (*ab*)*, dachshund (dac*)*, Distal-less (Dll*) and *spineless* (*ss*) double-stranded (ds)RNA at various concentrations were ablated. The numbers of *Tribolium* treated with the *ampicillin-resistance* (*amp*^*r*^) dsRNA are also included as a control. *For *ss*, the pattern of regeneration was different from the control *amp*^r^ dsRNA-injected regeneration; the regenerating antennae transformed into a leg.

### Dll is essential for regeneration of appendages

Injection of *Dll* dsRNA into day-0 sixth-instar larvae followed by leg ablation resulted in no regeneration of limbs. After one molt, all of the larvae (n = 18) were able to heal the wound around the area of leg ablation (Figure 2E; Table 4), similar to the *amp*^*r*^ dsRNA-treated animals. After two or more molts (Table 4), however, none of the *Dll* dsRNA-injected larvae were able to regenerate their ablated limbs (n = 18), even though the *amp*^*r*^ dsRNA-treated animals regenerated their limbs. All of the ablated leg segments, such as the femur, tibiotarsus and claw, were missing (Figure 2F). In the absence of Dll, only a round blastema-like structure was present on the ablated legs after two molts (Figure 2F), whereas in the control *amp*^*r*^ dsRNA-injected animals, all of the leg segments were re-established after two molts (Figure 2B). In addition, the morphology of the contralateral, unablated legs gradually became distorted with increasing number of molts, indicating that Dll is required to maintain leg identity, as previously reported [18]. Most larvae continued to molt as larvae and never metamorphosed. This inability to metamorphose was also observed previously in unablated larvae, so it was likely independent of wounding. The two adults that metamorphosized after more than two molts post *Dll* dsRNA injection did not regenerate any structures on their ablated side (Table 4; Figure 4I). Given that the blastema-like bump in these *Dll* dsRNA-injected larvae were prominent, Dll appears to be required for re-differentiation of the blastema-like bump into a functional leg, but not for the formation of the bump.

Similarly, day-0 sixth-instar larvae (n = 10) injected with 2 μg/μL *Dll* dsRNA followed by antennal ablation did not regenerate any structures after two molts (Table 5; Figure 5C). They formed rounded large blastema-like bumps that failed to grow back any of the antennal segments even after multiple supernumerary molts. Thus, Dll appears to be necessary for re-differentiation during larval appendage regeneration.

### Ab is required for blastema development

We next examined the effect of silencing *ab* during the larval stage on the regeneration of ablated larval legs. In all cases, after one molt, the wound healed and a smooth, rounded covering formed (Figure 2G). After the second molt, the legs had still not regenerated in the *ab* dsRNA-injected larvae (n = 19) (Table 4), and a small bump was observed (Figure 2H), indicating that little cell proliferation had taken place. Beyond the coxa, none of the larval leg segments were distinguishable. Although many larvae died prior to metamorphosis (n = 13), none of the animals that pupated after two larval molts regenerated pupal legs (n = 6). Similarly, the adults also failed to show any signs of leg regeneration: often no structures were visible at the ablated areas, or a tiny mass of unidentifiable tissue was observed with neither femur nor tibia regenerated (Figure 4E, arrows). In one particular adult that had developed a large bump after the second larval molt post injection, a malformed appendage developed (Figure 4F). The structure was sclerotized and exhibited normal pigmentation and bristle development. However, this appendage had one segment with several abnormal protrusions, as well as a pair of claw-like protrusions (Figure 4F) at the boundary between the coxa and the segment. The identity of the observed structure and the claw-like structures could not be determined, but the abnormal structure showed that Ab strongly influences growth/patterning during regeneration, in addition to regulating the growth of blastemas. This was in contrast to the unablated legs of the adults obtained from *ab* dsRNA-injected larvae, which were affected such that the tarsomeres were reduced in number (Figure 4F), but the femur and the claws at the tip of the tarsus remained unaffected, as reported previously [8,9]. Thus, although silencing of *ab* only affects tarsal segmentation during metamorphosis, it prevents legs from re-forming during regeneration.

The effect of removing larval Ab expression on the regeneration of antennae was variable. As with the *amp*^*r*^ dsRNA-injected control animals, sixth-instar larvae injected with 2 μg/μL *ab* dsRNA after one molt were able to heal their wounds and sometimes form blastema-like structures. After two molts, a few larvae (n = 4) were able to regenerate a new segment on the site of antennal ablation, whereas the rest (n = 7) did not regenerate any new structures (Figure 5D; Table 5). However, the majority of the animals that were injected with *ab* dsRNA and underwent antennal ablation died as larvae, as they got trapped in the old cuticle during the larval-larval molts. The head often became trapped in the old cuticle, and the larvae eventually died, presumably from starvation. Only two animals survived to metamorphose after one molt: one of them regenerated an antenna that resembled the unablated contralateral antenna, and the other became a pharate adult that did not exhibit any signs of antennal regeneration (not shown). The remaining larvae that survived past two molts (n = 11) were unable to properly eclose or metamorphose, and ultimately died as larvae. The observed deaths were not confined to larvae with ablated limbs: many unablated Ab-knockdown larvae also died prior to metamorphosis (not shown).

To see if we could obtain higher survival rates, we injected fifth-instar larvae (n = 9) with 0.5 μg/μL *ab* dsRNA. The degree of antennal regeneration was variable, as with the larvae injected with a higher concentration of dsRNA (Table 5). There were two larvae with regenerating antennae (developing new structures on the site of ablation) after two molts; one of these larvae molted successfully into an adult with a perfectly regenerated antenna that resembled the uncut control antenna. However, the remainder of the larvae (n = 7) did not show signs of antennal regeneration after two molts. In addition, decreasing the concentration to 0.5 μg/μL did not decrease the rate of larval mortality (8 larvae died after two molts). Thus, at either concentration, most *ab* dsRNA-injected larvae were unable to regenerate their ablated antennae after two molts (n = 7/11 for 2 μg/μL and n = 7/9 for 0.5 μg/μL; Table 5). Combined, these results indicate that Ab is required for blastema development during regeneration of both legs and antennae.

### Ss is not required for leg regeneration

When *ss* was silenced prior to leg ablation during the larval stage, both ablated legs began to regenerate by the second molt (n = 11 for 4 μg/μL and n = 7 for 2 μg/μL; Table 4; Figure 2J). These legs were similar to the regenerating legs of *amp*^*r*^ dsRNA-injected larvae and regenerated all of the larval leg segments. When smaller larvae were injected, larvae that molted three times had restored the morphology of leg segments, similar to *amp*^*r*^ dsRNA-injected larvae (compare Figure 3A and C). *ss-*knockdown without leg ablations resulted in the formation of adults with phenotypes similar to those obtained by Shippy *et al*. (2009) and Angelini *et al*. (2012) (Figure 4G and H) [9,10]. As described previously, the tarsomeres were fused and were shorter than those of the control *amp*^*r*^ dsRNA-injected adults. In the adults obtained from *ss* dsRNA-injected larvae followed by leg ablations, the regenerated legs were morphologically indistinguishable from the contralateral unablated legs (Table 4; Figure 4G and H). From these results, we infer that Ss is not necessary for larval leg regeneration.

When *ss* was knocked down in day-0 fifth- or sixth-instar larvae, all of the ablated antennae (n = 22) (Table 5) healed and formed rounded structures as seen in the control *amp*^*r*^-knockdown animals after the first molt (Figure 5A). After the second molt, all of the 2 μg/μL *ss* dsRNA-injected larvae (n = 21) developed leg-like structures in the place of a regenerated antenna (Figure 5E; Table 5). The regenerated antennae developed a claw and a bristle pattern resembling those seen in larval legs (Figure 5E, arrow and arrowhead). The unablated antennae remained unaffected and did not transform, indicating that Ss is only required when the larval antenna is regenerating. Such heteromorphic transformation of regenerating appendages was never seen with the knockdowns of any of the other transcription factors investigated here.

In the unablated antenna of *ss*-knockdown larvae, the antennae transformed partially into a leg-like structure during metamorphosis (n = 14). These animals have one large structure that appears to comprise a fusion of the femur, tibia and tarsomeres (Figure 6C; [10]). The adult structure on the ablated side had distinct leg-like structures with clearly distinguishable tibia and tarsomeres, in contrast to the contralateral unablated antennae, where the tibia and tarsomeres appeared to be fused. Presumably, the earlier transformation of the regenerating antenna into leg-like phenotype accounts for the more enhanced leg-like morphology observed in the *ss*-knockdown adult. Thus, similar to the effect of *dac* knockdown in regenerating legs, the antennal patterning defects in *ss* dsRNA-injected larvae were reflected in the adult antennal morphology. Taken together, our results indicate that Ss plays an important role in maintaining the identity of the antennae during larval antennal regeneration and metamorphosis.

### Knockdown verification

**Figure 7:**
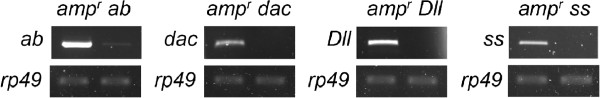
**Knockdown verification of double-stranded RNA (dsRNA)-injected larvae using semiquantitative polymerase chain reaction.** Day-0 sixth-instar larvae were injected with 2 μg/μL *ampicillin-resistance* (*amp*^*r*^)*, dachshund (dac*)*, Distal-less* (*Dll*)*, abrupt* (*ab*) and *spineless* (*ss*) dsRNA, and total RNA was isolated from day-2 seventh-instar larvae. This RNA was converted to cDNA, and gene-specific primers were used to amplify the gene products. *amp*^*r*^ dsRNA-injected larvae served as controls. The cycle numbers were as follows: *ab* = 38; *dac* = 42; *Dll* = 39; *ss* = 40; *rp49* = 34.

We performed semiquantitative PCR to determine the gene expressions of *dac*, *Dll*, *ab* and *ss* in response to dsRNA injections. We confirmed that gene expression was knocked down through dsRNA injection by comparing the expression in *amp*^*r*^ dsRNA-injected animals with that in the dsRNA-injected animals (Figure 7). Compared to the control larvae, the target gene expression was reduced in larvae injected with dsRNA of the respective genes (Figure 7). Thus, we established that the phenotypes shown in this study were the effects resulting from the knockdown of each gene.

## Discussion

In this study, we examined the roles of limb-patterning genes during embryonic development and regeneration, and the degree of developmental coupling between larval and adult limb-patterning (Table 2). We found that *dac-*knockdown embryos developed legs with abnormally shortened medial segments, while the antennae developed normally. In addition, silencing of *dac* resulted in loss of the medial segments of the regenerating legs; this altered phenotype was carried over to the adult leg morphology. However, no major effects were detected in the regenerating antennae. Knockdown of *Dll* completely inhibited regeneration of the ablated appendages, although a prominent blastema-like structure formed. *ab-*knockdown embryos exhibited a unique scrunched phenotype throughout their entire body, including their antennae and legs; however, each of the segments of these appendages was distinguishable. These embryos also displayed abnormal bristle patterning on the dorsal tergites. Ab was found to be essential for blastema growth/development. *Ss*-knockdown larvae were able to regenerate their limbs, but a heteromorphic transformation of the larval antenna to a leg was observed when the antennae were removed. This larval heteromorphic effect was also carried over to the adult stage.

### Dac and Ss function similarly during regeneration and embryonic development

Although *dac* dsRNA-injected *Tribolium* were able to regenerate both the larval legs and antennae after two molts (Tables 4 and 5), knockdown of Dac expression disrupted the patterning of the medial segments in the regenerating legs (Table 2; Figure 3B). Our results indicate that Dac acts specifically in the legs to pattern the medial segments during regeneration, which is consistent with past studies of Dac in cricket leg regeneration [34]. In the antennae, Dac seems to have no apparent role during larval regeneration, and the regenerated adult antennae were indistinguishable from the unablated antennae (Figures 5B and 6B). Similar effects of patterning were observed in the *dac-*knockdown embryos: in embryos affected by *dac* dsRNA, the antennae developed normally, but the legs had a shortened femur-tibiotarsus segment, akin to the regenerated larval legs of *dac*-knockdown larvae. These findings are consistent with previous studies, which have determined that *dac* is most strongly expressed in the legs and less so in the antennae [15,35], suggesting that the role of Dac in patterning the medial segments of the antenna is largely confined to metamorphosis [8], and not regeneration or embryonic development. Thus, Dac functions similarly during larval appendage regeneration and embryonic development (Table 2).

Legs regenerated normally when the expression of *ss* was knocked down. However, knockdown of *ss* in the fifth or sixth instars affected the regeneration of the antennae. The ablated antenna underwent a heteromorphic transformation and developed into a structure that resembled a leg. A similar transformation of an antenna to a leg was also observed in embryos [10,29]. The embryonic phenotypes resulting from dsRNA treatment suggest that Ss is not involved in the embryonic patterning of legs but plays a key role in specifying antennal identity. In contrast, during metamorphosis, Ss is required for patterning the distal segments of the developing adult legs [8-10,25]. Taken together, the functions of Ss and Dac during regeneration are similar to those seen during embryonic, rather than metamorphic, development (Table 2).

### Dll is required for initiation of leg outgrowth during regeneration

None of the animals injected with *Dll* dsRNA were able to regenerate any structure distal to the site of ablation in either the legs (Tables 2 and 4; Figure 2E, 2F and 4I) or the antennae (Tables 2 and 5; Figure 5C). For all of the *Dll*-knockdown animals, a large bump formed on the ablated sites and remained throughout the molts, indicating that the bump is able to grow. However, no appendages ever differentiated from this bump, and the legs were absent from the adult (Figure 4I). At this point, we do not have any blastema markers so we are unable to determine the nature of this bump. However, given the previous finding that Dll is required to maintain the appendage identity [18], Dll may be required for re-differentiation of cells inside this bump.

### Ab is required for initiation of regeneration

Our study shows that Ab plays an essential role in limb regeneration. Beyond the formation of a small bump, appendage regeneration appeared to be stunted in *ab-*knockdown animals. This indicates that Ab may be involved in cell proliferation of blastema cells during leg regeneration. The effects of *ab* knockdown on antenna regeneration, though less definitive given that the antennae of a few larvae regenerated after two molts (Table 5), also indicated that Ab is required for the initiation of antennal regeneration.

Unlike the effect of silencing *ab* on regeneration, Ab was not required for the initial allocation and subsequent formation of either antennae or legs during embryonic development (Table 2). During metamorphosis, the tarsomeres became fused in *ab*-knockdown animals; however, the rest of the leg was unaffected. Thus, while Ab is involved in the distal patterning of adult legs during normal development, Ab appears to play a distinct role during regeneration by affecting the development and growth of the blastema (Table 2). Together, these data indicate that the role of Ab during regeneration is separate from its embryonic and metamorphic function in normal development.

The *ab*-knockdown phenotype is similar to that obtained when Wnt-1 expression is silenced. When Wnt-1 is silenced, the blastema fails to grow, and the legs are never able to regenerate, whereas the amount of antennal regeneration is variable between individuals [14]. Thus, Ab and the Wnt signaling pathway may influence the same process during regeneration and may potentially interact. It is interesting that both Wnt-1 and Ab knockdown animals form rudimentary wings during metamorphosis (unpublished observations and [14]).

### Re-patterning phase of limb regeneration relies on taxon-specific regulation

Limb regeneration in most metazoans, including *Tribolium*, can be classified into four general stages: wound healing, blastema formation, blastema cell proliferation, and re-patterning [14,36-38]. Although we have found that both Dac and Ss are necessary for the re-patterning of certain appendages in *Tribolium*, these genes most likely are not conserved throughout the Metazoa, because they are specific to the patterning of arthropod appendage segments. Similarly, genes that are necessary for normal limb patterning and re-patterning in vertebrates, like fibroblast growth factor (FGF) [39-46], appear not to play significant roles, at least during the earliest stages of *Tribolium* limb re-patterning [47]. Thus, while certain factors appear to play roles in regeneration across all regenerating metazoans [14,47-52], genes involved in re-patterning are unlikely to be a part of any conserved gene regulatory networks underlying limb regeneration. Rather, it appears that once the regeneration pathway is activated, taxon-specific embryonic patterning genes are used to re-pattern the appendages. Whether or not the first steps of limb regeneration, such as wound healing and blastema formation, are evolutionarily conserved processes remains to be seen.

### Developmental uncoupling between larval and adult morphologies

An unresolved issue in holometabolous insect development and evolution is the developmental continuity between larval legs and adult legs. At the cellular level, there appears to be a smooth transition between the larval appendages and adult appendages in most insects except in the case of lepidopterans, where the adult appendages actually arise from set-aside imaginal cells that reside within the larval legs and proliferate during the final instar [4]. What is less clear is how patterning of larval appendages affects adult appendages. Our study shows that larval appendage patterning is inherited by the adult appendages. For example, when Dac is silenced in the regenerating larval legs, the medial segments are deleted, and this patterning alteration is also observed in the adult stage. While unablated adult legs are also affected, the regenerated leg is more severely affected. This implies that either the fates of the presumptive adult cells are already specified by the time they pupate or the number of cells present in the larval appendage constrains the adult leg morphology. Regardless of the mechanism, our study indicates that larval appendage patterning might pose a developmental constraint on the adult appendages. Thus, while the evolution of metamorphosis allowed the generation of two distinct morphs, the larva and the adult, which are each adapted to a different habitat, the two life-history stages may constrain each other, at least in some holometabolous insects, such as beetles.

The heteromorphic transformations seen in *ss* knockdown animals have interesting implications for understanding the developmental basis for naturally occurring heteromorphic transformations. The theory of homeotic transformation was first developed by Bateson (1894) to describe the replacement of one structure, such as the antennae, with a completely different one, such as the tarsus of a leg [53]. Though at first believed to be isolated examples of developmental anomalies, various studies have demonstrated that heteromorphic transformations could be experimentally induced by physical mutilation during late development [54,55]. For example, injuring the antennae of a sawfly, *Cimbex axillaris*, can cause the heteromorphic transformation of the adult antenna into a leg [55]. However, the molecular basis of postembryonic heteromorphic transformation of an appendage has not been well-characterized. Our study demonstrates that given the continuity of larval and adult stages, alteration of a single transcription factor, such as Ss, during a specific time period of larval life can induce the homeotic transformation of the regenerating larval antennae and subsequently influence the adult antennal identity.

Compartmentalization of developmental processes has been proposed to be a major source of evolvability for different stages of complex life cycles [1]. In insects with larval limbs that transform into adult limbs, developmental coupling between larval and adult stages might constrain evolutionary change. Many derived insect species have independently evolved imaginal discs that have enabled complete separation of larval and adult life stages [1,2,56]. Thus, in addition to promoting rapid growth in an organism with a short life cycle, the evolution of imaginal discs may serve as a final step towards complete dissociation of larval and adult structures.

## Conclusions

Our study indicates that larval limb morphology is linked to the adult limb patterning in *Tribolium*. We propose that the evolution of free-floating imaginal discs may have been a key step towards to completely removing the developmental constraint that existed between the larval and adult phenotypes. Thus, the evolution of insects is characterized by a gradual loss of developmental coupling between the juvenile and adult stages.

## Abbreviations

Ab: Abrupt; ampr: Ampicillin-resistance gene; BTB: Bric-a-brac tramtrack broad complex; dac: Dachshund; Dll: Distal-less; dsRNA: Double-stranded RNA; FGF: Fibroblast growth factor; PBS: Phosphate-buffered saline; PCR: Polymerase chain reaction; PD: Proximal-distal; RNAi: RNA interference; ss: Spineless; ssRNA: Single-stranded RNA.

## Competing interests

The authors declare that they have no competing interests.

## Authors’ contributions

AKL participated in the design of the study, carried out the molecular genetic studies and drafted the manuscript. CCS participated in the design of the study, carried out the molecular genetic studies and drafted the manuscript. ERK carried out the molecular genetic studies and helped to draft the manuscript. YS participated in the design and coordination of the study, carried out the molecular genetic studies and helped to draft the manuscript. All authors read and approved the final manuscript.

## Supplementary Material

Additional file 1 **Effects of *Dll *and *ss* knockdown on embryonic development.** (**A**,**D**) Whole-body image of the embryo injected with *Dll* (**A**) and *ss* (**D**) dsRNA. (**B**) Head of a *Dll* knockdown embryo. Arrowheads point to the antennae that lacked the distal flagellum. (**C**) Legs of *Dll*-knockdown embryo that lacked the distal portions (arrowheads). Close-up image is shown to the right. (**E**) Head of a *ss*-knockdown embryo. Arrowheads indicate the antennae that had transformed into legs. (**F**) Legs were unaffected by *ss* knockdown. *Dll*, *Distal-less*; *ss*, *Spineless*; dsRNA, double-stranded RNA. Click here for file
