# Supplementary figures and images for: Developmental coupling of larval and adult stages in a complex life cycle: insights from limb regeneration in the flour beetle, Tribolium castaneum
Source: EvoDevo. 2013 Jul 4;4:20. doi: 10.1186/2041-9139-4-20 (PMC3711857; doi:10.1186/2041-9139-4-20)

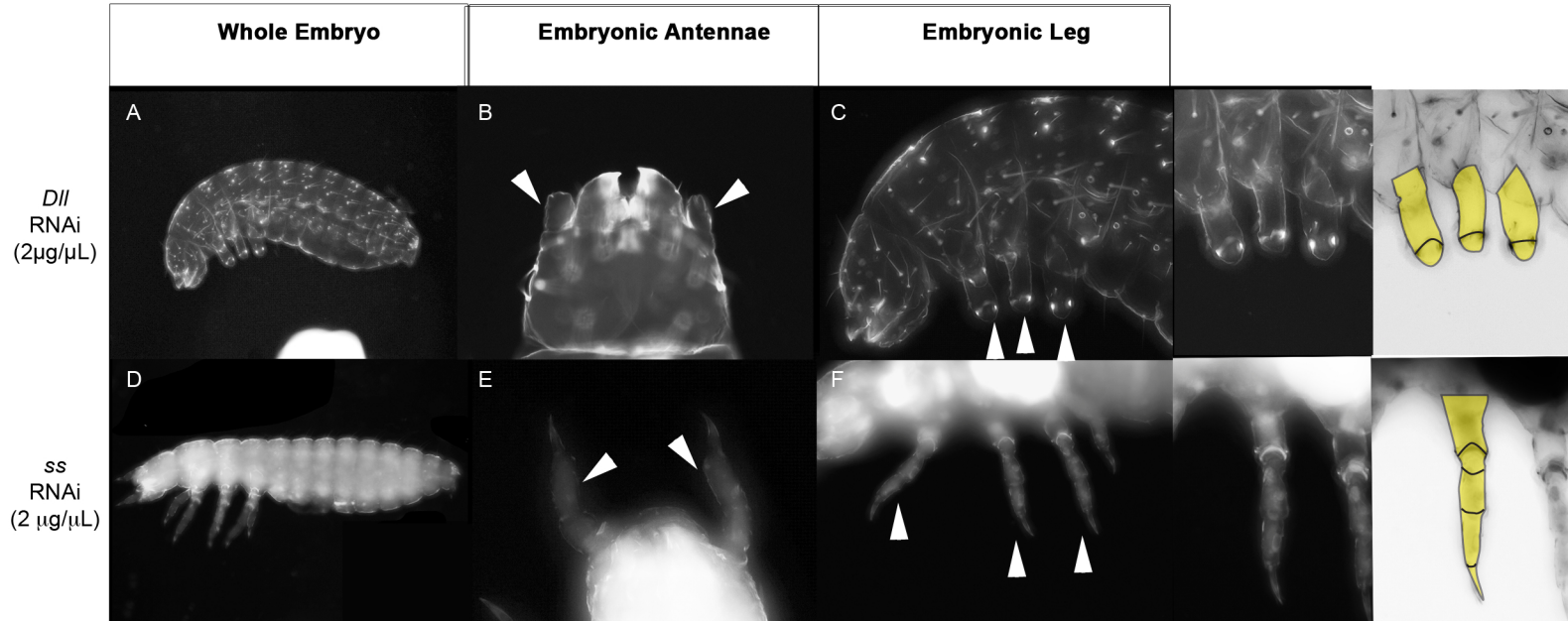

Supplement: Additional file 1 — Effects of Dll and ss knockdown on embryonic development. (A,D) Whole-body image of the embryo injected with Dll (A) and ss (D) dsRNA. (B) Head of a Dll knockdown embryo. Arrowheads point to the antennae that lacked the distal flagellum. (C) Legs of Dll-knockdown embryo that lacked the distal portions (arrowheads). Close-up image is shown to the right. (E) Head of a ss-knockdown embryo. Arrowheads indicate the antennae that had transformed into legs. (F) Legs were unaffected by ss knockdown. Dll, Distal-less; ss, Spineless; dsRNA, double-stranded RNA. [file 2041-9139-4-20-S1.pdf]
